# A Case of Urinary Calculus, Attended with Peculiar Circumstances and Treated by Lithotrity

**Published:** 1851-04

**Authors:** L. A. Dugas

**Affiliations:** Prof. of Surgery in the Medical College of Georgia


Art. III.—*A Case of Urinary Calculus, attended with peculiar circumstances and treated by Lithotrity.* By L. A. Dugas, M. D., Prof. of Surgery in the Medical College of Georgia.

The following case is reported because of certain peculiar features presented duing its progress. The patient, Mr. John L. B., of Hall county, Ga., is 30 years of age, was kindly directed to my care by Dr. Richard Banks, the distinguished surgeon of Gainesville, and arrived here on the 5th of February last. Having suffered from early childhood with phymosis and an almost complete closure of the orifice of the prepuce, (which he believes was congenital,) the difficulty of voiding his urine caused this to distend the prepuce into a considerable bag, to accumulate enormously in the bladder, to stagnate in the pelvis of the kidneys, and to induce very great impairment of the general health. The preputial orifice was so small as not to admit, without much difficulty, the introduction of a knitting needle; the urine was therefore never passed off in a jet, but the patient was subjected to all the inconvenience of a continual stillicidium; he had frequent and violent attacks of nephritic pains, attended with protracted chills, fevers, and the usual concommitants of retention of urine. Yet it was not until the 20th year of his age that he sought professional aid and was circumcised by Dr. Banks. From that time his health improved rapidly; but he continued subject to occasional paroxysms of severe nephritic pains, which now became confined to the left side. These pains would extend down along the course of the ureter and continue one or more days, leaving him in a debilitated state, from which he would, however, soon recover. He is not aware of ever having passed gravel or anything like calculous matter, although his urine would sometimes present a very copious sediment.

This state of things continued until the middle of April last, when, although in good health and not having had any nephritic pain for about three months, he felt a calculus drop into his bladder. Attending to his usual avocations, he stepped out to urinate, did so without any difficulty whatever, and when in the act of buttoning up his garment, distinctly felt something fall into the bladder.

He immediately mentioned the fact to a friend, and added that "it must be a stone, for its fall produced a sensation like that of a buck-shot allowed to drop into a bag." A few hours afterwards, on again attempting to urinate, the stream was suddenly arrested by the engagement of the calculus in the urethra—the sensation being so distinct that he instinctively carried his hand to the perineum in order to force it out—but in vain;—and the same difficulty has ever since attended his micturition. These details are given as establishing conclusively the facts that he did know the *precise moment* at which the stone came into the bladder, and that this occurred so late as about *three months after the last nephritic attack.* He has experienced no pain whatever about the kidney since that. In May he was sounded by Dr. Banks, who readily detected the stone.

On the arrival of Mr. B. here, I examined him, detected the calculus, found it to be small and determined to crush it as soon as circumstances would permit. The patient was directed to use dilating bougies, to remain quiet, to drink freely of slippery elm tea and super carbonate of soda, and to take a hip bath every night. In a week he was found to be sufficiently prepared, and (on the 12th of February) the operation was performed with Heurteloup's "*brise pierre*," as modified by Charriere. The bladder being filled with tepid water, the calculus was readily seized and crushed three times, without pain. A few fragments were passed off with the water and others during the night with the urine. On the following day, finding the patient very comfortable, without any symptoms of irritation, and very anxious to get home as soon as possible, I again introduced the instrument and crushed the remaining fragments, sufficiently to allow them all to be passed out during the night. He now expressed himself "entirely relieved, and feeling like a new man." The baths, etc., were continued, and on the 16th February, I explored the bladder carefully, without being able to detect any vestige of the stone. The patient was therefore discharged.

The dimensions of the stone were accurately ascertained by the crushing instrument to be about one inch in length and half an inch in thickness. Professor Means having kindly subjected some of the fragments to analysis, informs me that they consisted of Oxalate of Lime. The stone was exceedingly hard, and tested to the uttermost the fine temper imparted to the metal by Charriere's unrivalled skill.—*South. Med. and Surg. Jour.*

Remarks.—This certainly presents "certain peculiar features," both in anatomy and surgery, and we are utterly at a loss to understand some of them. The fault may be ours, but there can be no wrong in stating the difficulties.

1st.—It is somewhat remarkable that a phymosis should have created so great a resisting power in the prepuce as to dilate even the ureters. This strikes us as a very remarkable peculiarity. The wonder is increased considerably when we find that notwithstanding the *ureters* were thus dilated so as to permit the passage of a stone of novel dimensions, the *urethra*, which should have synchronised liberally in the dilatation of the ureters, was so little inclined towards anything of the kind, that it stopped the stone which had fallen through the ureter! The extravagant dilatation of the ureter is inexplicable; but, assuming the claim as a fact, the dilatoriness of the urethra is rather marvellous.

2d.—The statement of the patient that he "heard something drop," and therefore knew the exact moment of the entrance of the calculus into the bladder, seems to have made a profound impression upon Professor Dugas, for he unhesitatingly gave credence to the statement. The patient may be excused for thinking that a calculus could fall from the ureter into the bladder, but we have some difficulties in our faith. The ureters enter the *bas fond* of the bladder, very obliquely, and a stone would have to fall *up* in falling from the ureter into the bladder. And then when we remember the pathological truths of Mr. Aldridge, which seem to show that the oxalate of lime is not secreted in the kidneys, when we remember that there is no kind of evidence that the ureters in this case were dilated even in the slightest degree, aud that the passage of a mulberry calculus through the ureter would have made a man feel a multitude of other things besides the falling of the calculus, we must remember that we have before us what may be called the difficulties of faith.

3d.—We feel some difficulty about the dimensions of the calculus. We have seen between two and three hundred specimens of calculi, and have heard from various other collections, and we have neither seen nor heard of any calculus, except this one in Georgia, that was just one inch in length, and a half inch in thickness. These dimensions are such a wide departure from that uniformity of proportion found in calculi, that we think there must be some mistake in Professor Dugas's measurements. There must be a want of accuracy. Did it not strike the Professor that the growth of his specimen was altogether too rapid for a case of oxalate of lime calculus? There seems to us a wonderful celerity in every branch of this case.

4th.—The calculus in this case was "oxalate of lime," and the stone was crushed with Heurteloup's "*brise pierre*" at two sittings, on two consecutive days, and the fragments were allowed to be passed off during the night. This is certainly the most remarkable achievement yet effected by Heurteloup's instrument. It is enough to excite the envy of Civiale, and put an end to the lateral operation. If a calculus of oxalate of lime, one inch long, and a half inch thick can be utterly crushed in two sittings, in two successive days, so that no vestige of it is left, what apology can there be for cutting instruments for lithotomy? We have seen various efforts with Heurteloup's instrument, and have been sometimes surprised with the result, but this success in breaking down, in two sittings, a stone of oxalate of lime, of the size of the one recorded by Professor Dugas, certainly takes the lead of all achievements we know of in lithotrity. We have seen vesicle stones of oxalate of lime removed by the lateral operation after lithotrity had failed, and in which the most persistent efforts with the drill for many sittings had failed to make any more impression than if it had been used on a piece of Syenite. But if the improved apparatus of Heurteloup can break up at two sittings, a mass of oxalate of lime, and remove it entirely in two days, lithotrity is making rapid strides, and M. Roux is an accredited prophet, when he says: "lithotrity has assumed her function, and no surgeon hereafter will attain sufficient experience to reach the highest degree of adroitness in lithotomy."

We suppose these new claims of lithotrity will come before the American Medical Association, and if they receive the endorsement of that body, we may expect to see renewed evidences of the envy felt by European surgeons for the rising reputation of American Surgery, and we shall hear them again denouncing American surgeons for a proneness to exaggeration. B.

